# Multiple steps characterise ventricular layer attrition to form the ependymal cell lining of the adult mouse spinal cord central canal

**DOI:** 10.1111/joa.13094

**Published:** 2019-10-31

**Authors:** Marco A. Cañizares, Aida Rodrigo Albors, Gail Singer, Nicolle Suttie, Metka Gorkic, Paul Felts, Kate G. Storey

**Affiliations:** ^1^ Division of Cell & Developmental Biology School of Life Sciences University of Dundee Dundee UK; ^2^ Centre for Anatomy & Human Identification University of Dundee Dundee UK

**Keywords:** bone morphogenetic protein signalling, cell cycle, central canal, dorsal collapse, ependymal cells, floor plate, FUCCI reporter, roof plate, Sonic hedgehog signalling, spinal cord development, ventral dissociation, ventricular layer

## Abstract

The ventricular layer of the spinal cord is remodelled during embryonic development and ultimately forms the ependymal cell lining of the adult central canal, which retains neural stem cell potential. This anatomical transformation involves the process of dorsal collapse; however, accompanying changes in tissue organisation and cell behaviour as well as the precise origin of cells contributing to the central canal are not well understood. Here, we describe sequential localised cell rearrangements which accompany the gradual attrition of the spinal cord ventricular layer during development. This includes local breakdown of the pseudostratified organisation of the dorsal ventricular layer prefiguring dorsal collapse and evidence for a new phenomenon, ventral dissociation, during which the ventral‐most floor plate cells separate from a subset that are retained around the central canal. Using cell proliferation markers and cell‐cycle reporter mice, we further show that following dorsal collapse, ventricular layer attrition involves an overall reduction in cell proliferation, characterised by an intriguing increase in the percentage of cells in G1/S. In contrast, programmed cell death does not contribute to ventricular layer remodelling. By analysing transcript and protein expression patterns associated with key signalling pathways, we provide evidence for a gradual decline in ventral sonic hedgehog activity and an accompanying ventral expansion of initial dorsal bone morphogenetic protein signalling, which comes to dominate the forming the central canal lining. This study identifies multiple steps that may contribute to spinal cord ventricular layer attrition and adds to increasing evidence for the heterogeneous origin of the spinal cord ependymal cell population, which includes cells from the floor plate and the roof plate as well as ventral progenitor domains.

## Graphical abstract

This study identifies key steps that characterise attrition of the spinal cord ventricular layer as it is re‐modelled to form the ependymal cell lining of the adult central canal – these include localised dorsal and ventral cell re‐arrangements, reduced cell proliferation and altered dorsal and ventral signalling.

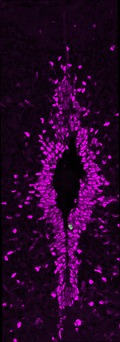

## Introduction

The ependymal cells that form the lining of the central canal in the adult mammalian spinal cord constitute a largely quiescent stem cell niche (Adrian & Walker, 1962; Kraus‐Ruppert et al. 1975; Sabourin et al. 2009; Alfaro‐Cervello et al. 2012). These cells can be induced to re‐enter the cell cycle in response to extrinsic stimuli, including mechanosensory stimulation (Shechter et al. 2011), physical exercise (Krityakiarana et al. 2010), inflammation (Chi et al. 2006; Danilov et al. 2006) and injury (Adrian & Walker, 1962; Frisen et al. 1995; Johansson et al. 1999; Meletis et al. 2008; Barnabe‐Heider et al. 2010; Li et al. 2016, 2018). Moreover, most of these stimuli appear to promote the generation of new neurons and glial cells, consistent with *in vitro* studies of the differentiation potential of the spinal cord ependymal cell population (Weiss et al. 1996; Johansson et al. 1999; Li et al. 2016; Meletis et al. 2008; Sabourin et al. 2009). However, following spinal cord injury, these ependymal cells proliferate and migrate to the lesion site, but here differentiate into only glia (Barnabe‐Heider et al. 2010; Li et al. 2016, 2018; Meletis et al. 2008; Martens et al. 2002). These cells then contribute to scar tissue, many becoming astrocytes which reduce inflammation, but chronically inhibit axonal re‐growth (Warren et al. 2018), whereas others differentiate into oligodendrocytes, which can promote survival of nearby neurons and help to maintain the integrity of the injured spinal cord (Sabelstrom et al. 2013). Together, these findings indicate that changes in environment determine the behaviour and differentiation of spinal cord ependymal cells. Importantly, this is a heterogeneous cell population and the precise identity of cells with neural stem cell abilities has yet to be determined. This activity of spinal cord ependymal cells is also distinct from that of ependymal cells lining the brain ventricles, where instead the neural stem cells constitute a distinct sub‐ependymal cell population (Mirzadeh et al. 2008; Shah et al. 2018; Lim & Alvarez‐Buylla, 2016). In the healthy animal, adult spinal cord ependymal cells carry out specialised functions, including homeostatic regulation of cerebrospinal fluid (CSF) composition and acting as a barrier between CSF and the spinal cord parenchyma (reviewed in del Bigio, 1995; Bruni, 1998). However, despite these significant roles in the injured and healthy spinal cord, little is known about how spinal cord ependymal cells arise and how the central canal is formed during development.

One aspect of central canal formation involves attrition of the progenitor cell population that constitutes the ventricular layer of the embryonic spinal cord (Fu et al. 2003; Shibata et al. 1997; Yu et al. 2013). This remodelling process includes a striking morphological phenomenon known as dorsal collapse, which mediates a pronounced reduction of the dorsal ventricular layer in a range of mammals (Barnes, 1883; Bohme, 1988; Elmonem et al. 2007; Sevc et al. 2009; Sturrock, 1981). However, the changes in cell behaviour that underlie this critical event are poorly understood. In contrast, the earlier dorso‐ventral subdivision of the developing spinal cord has been well‐characterised. This involves signals emanating from the roof plate located at the dorsal midline, including bone morphogenetic protein (BMP) and Wnt, and the floor plate at the ventral midline (Sonic hedgehog, Shh), which act in opposition to specify distinct neural progenitor cell populations along the dorso‐ventral axis (Jessell, 2000; le Dreau & Marti, 2012; Ulloa & Briscoe, 2007). This involves regulation of homeodomain and other transcription factors, which act in combination to define neuronal subtype specific progenitors (Lee & Pfaff, 2001). Key transcription factors include *Pax6*, which is initially expressed broadly and is involved in motor neuron progenitor specification, and *Nkx6‐1*, which distinguishes V2 interneuron progenitors. Importantly, the continued expression of *Pax6* and *Nkx6‐1* in the adult ependymal cells has led to the notion that these cells derive from this earlier population of ventral neural progenitors (Fu et al. 2003; Yu et al. 2013). It is apparent that this ventral region of the ventricular layer is also reduced over time and this may be associated with the switch from neurogenesis to gliogenesis between E11.5 and 12.5 and, ultimately, the migration of glial cells out of this layer (Deneen et al. 2006; Stolt et al. 2003; reviewed in Laug et al. 2018). As the cells that make up the emerging central canal become separated from the most dorsal and ventral regions of the spinal cord, its formation may additionally involve the remodelling of these specialised cell populations. Indeed, dorsal collapse coincides with elongation of processes from nestin‐expressing cells from the roof plate, which ultimately integrate into the walls of the adult central canal in mammals (Bohme, 1988; Sevc et al. 2009; Xing et al. 2018; Shinozuka et al. 2019; Ghazale et al. 2019) and fish (Kondrychyn et al. 2013). It is also possible that a similar ventral reorganisation takes place and that this may account for the apparent inclusion of some floor plate cells in central canal (Khazanov et al. 2017).

Here, we describe sequential cell rearrangements associated with the attrition of the ventricular layer as the spinal cord matures during mid to late mouse embryogenesis. We determine whether changing patterns of cell proliferation and/or programmed cell death may contribute to this process. We further evaluate the contribution of floor plate cells to the wall of the forming central canal, assessing this in the context of changing expression patterns of genes and proteins associated with key dorsal and ventral signalling pathways.

## Methods

### Animals

Mice were either wild type CD1 or C57BL/6J strains (Charles River). Embryos and spinal cords from Fucci2a transgenic mice (Mort et al. 2014) were provided by Professor Andrew Jackson (MRC HGU, University of Edinburgh) and embryos and spinal cords from Shh‐green fluorescent protein (GFP) (Chamberlain et al. 2008) and gli‐binding sites (GBS)‐GFP (Balaskas et al. 2012) transgenic mice were provided by Dr James Briscoe (Francis Crick Institute). For timed matings, the morning of the plug was considered E0.5. All animal procedures were approved by the UK Government Home Office and in accordance with European Community Guidelines (directive 86/609/EEC) under project licence number 6004454.

### Immunofluorescence

From embryos, the inter‐limb (thoracic) spinal cord tissue was dissected in ice‐cold phosphate‐buffered saline (PBS), fixed for 2 h at 4 °C in 4% paraformaldehyde (PFA), rinsed several times in PBS and cryopreserved in 30% sucrose/PBS at 4 °C overnight. This tissue was embedded in 1.5% Luria‐Bertani (LB) agar/5% sucrose, again cryopreserved in 30% sucrose/PBS overnight, frozen on dry ice, and either cryosectioned in 20‐μm sections or stored at −20 °C for later use. To obtain adult spinal cords, 10‐ to 24‐week old mice were deeply anaesthetised with an overdose of pentobarbital (Euthatal) or isoflurane and then transcardially perfused with ice‐cold PBS followed by 4% PFA. Spinal cords were quickly dissected out and post‐fixed in 4% PFA for 2 h at 4 °C and processed as above. Standard procedures were used for immune fluorescence, with the following exceptions. To detect phosphorylated SMAD1/5, all solutions were supplemented with PhospoStop phosphatase inhibitor (Roche, Cat. # 4906837001) and sections were dehydrated with methanol to improve permeability. To detect pH3, sections were exposed to citrate buffer (pH 6) at 95 °C for 20 min. In all cases, sections were placed in blocking buffer (2–10% heat‐inactivated donkey or goat serum and 0.3–1% Triton X‐100 in PBS) for 30 min at room temperature and incubated at 4 °C overnight with primary antibodies diluted in blocking buffer. Lists of primary and secondary antibodies used are provided in Supporting Information Table S1. Nuclear counterstaining was achieved with DAPI diluted in PBS (1 : 1000) and incubated for 5 min at room temperature (RT). Slides were mounted with ProLong® Gold Antifade Mountant (Thermo Fisher Scientific, P36930) and images were captured on a DeltaVision or Leica TCS SP8 confocal microscope system. Images shown in all the figures are representative of sections analysed.

### RNA *in situ* hybridisation

Inter‐limb spinal cord tissue was dissected, fixed and processed as described above, but cryosectioned at 50 μm. Adult mouse spinal cords (24–40 weeks old) were also prepared as above but post‐fixed overnight in 4% PFA at 4 °C. Plasmids, restriction endonucleases and RNA polymerases used for the generation of digoxigenin‐labelled probes (Roche Applied Science) are provided in Supporting Information Table S2. Probes were diluted 1 : 100 in hybridisation buffer (50% deionised formamide, 5X SSC, 0.08% Tween 20, 50 μg mL^−1^ heparin, 1 mg mL^−1^ t‐RNA, 5 mm ethylenediaminetetraacetic acid (EDTA), 0.1% CHAPS, 0.02 g mL^−1^ Boehringer blocking reagent) and slides incubated at 65 °C in a chamber humidified overnight. Sections were washed in post‐hybridisation buffer (1X SSC, 50% formamide) at 65 °C for 15 min three times, followed by a 1 : 1 post‐hybridisation buffer/TBS‐0.1% Triton X‐100 (post‐hyb) wash at 65 °C for 20 min and blocked in 2% Boehringer blocking reagent, 20% heat‐inactivated sheep serum in post‐hyb for 1 h. Probe was detected using alkaline phosphatase‐labelled anti‐digoxigenin antibody (1 : 1000, Promega, 11093274910) in blocking buffer at 4 °C overnight. Sections were then washed with in post‐hyb and equilibrated for 10 min in NTMT buffer (100 mm NaCl, 100 mm Tris HCl pH 9.5, 50 mm MgCl_2_, 1% Tween 20). Finally, sections were incubated with alkaline phosphatase substrates nitro blue tetrazolium (NBT) and 5‐bromo‐4‐chloro‐3‐indolyl phosphate (BCIP) in NTMT buffer to reveal signal, washed in PBS‐0.1% Triton X‐100 and post‐fixed in 4% PFA at 4 °C and washed again in PBS‐0.1% Triton X‐100 and slides mounted with ProLong® Gold Antifade Mountant. Images were acquired on a Leica DMRB microscope fitted with a Nikon D7100 camera.

### TdT‐UTP nick end labelling (TUNEL) assay

The inter‐limb spinal cord tissue from CD1 mouse embryos was dissected and fixed at 4 °C for 2 h or overnight in 4% PFA. TUNEL assay was performed on 20‐μm‐thick cryosections using ApopTag® Peroxidase In Situ Apoptosis Detection Kit (Merck Millipore, Cat. # S7100) according to the manufacturer's instructions.

### Tissue measurements and cell counts

Images were processed using fiji and assembled using Adobe photoshop and illustrator software. Measurements of the dorsal‐ventral length and apical‐basal width of the ventricular layer were determined using Deltavision softworx software. The widest point was taken as the measure for width. The Cell Counter plugin in fiji was used to count SOX2‐expressing cells labelled with phospho‐histone H3 (pH3) or proliferating cell nuclear antigen (PCNA) and the same approach was used to count fluorescent cells in tissues from the FUCCI mouse line.

### Statistical analysis

All the results were summarised as mean ± standard error of the mean (SEM). A two‐sided, unpaired Student's *t*‐test with pooled/equal variance was used to test whether the differences in the means of two groups were statistically significant. A *P*‐value below 0.05 was considered to be statistically significant. The number of samples studied in each experiment is mentioned in their respective figure legends (see Supporting Information Tables S3–S6). No statistical method was used to determine the sample size.

## Results

### Changes in tissue dimensions and local cell rearrangements are associated with ventricular layer attrition during formation of mouse spinal cord central canal

**Figure 1:**
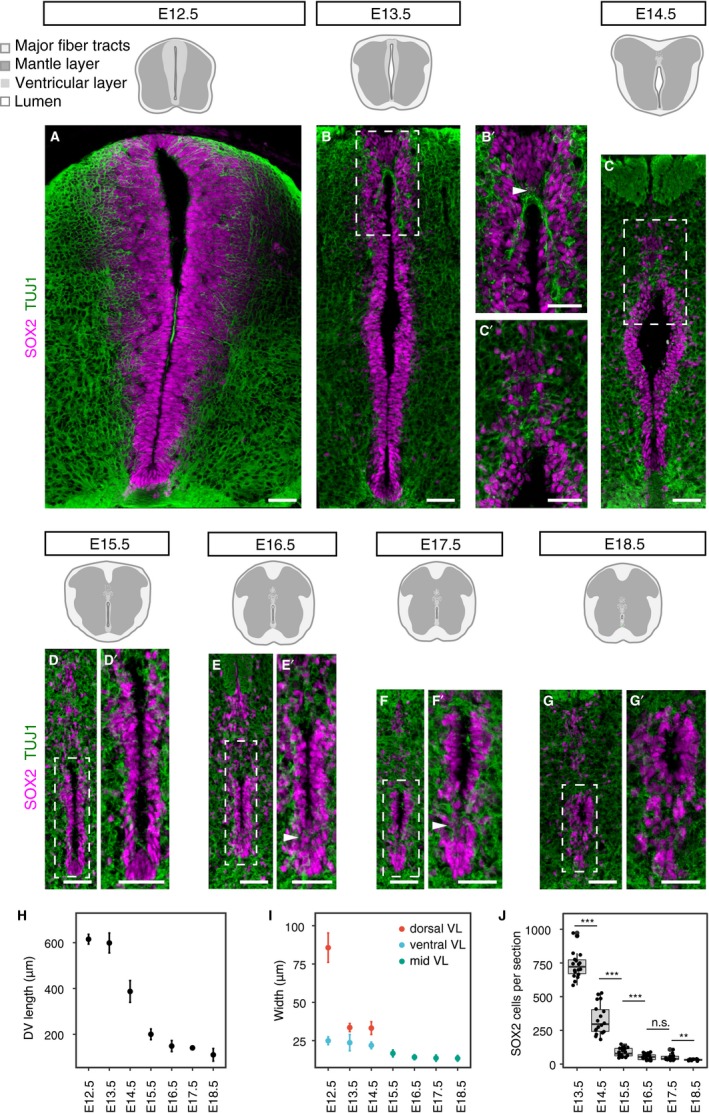
Changing ventricular layer dimensions during mouse spinal cord development. (A–G’) Immunofluorescence for SOX2 (magenta) and TUJ1 (green) reveals the changes in the organisation of the ventricular layer and the surrounding mantle layer (black dashed boxes) at the indicated stages. Higher magnification images of white dashed boxes from E13.5 to E18.5 are shown in B’, C’, D’, E’, F’ and G’, respectively. Note that some of the ventral‐most SOX2‐expressing cells appear to dissociate from the ventricular layer at later stages (arrowheads in E’ and F’). (H–I) Ventricular layer length (H) and width (I) at indicated stages (at least nine sections, *n* = 3 embryos for each stage). Widest horizontal distance across the dorsal region of the ventricular layer (dorsal VL) and adjacent to the floor plate (ventral VL) was measured. From E15.5 the width of the ventricular layer becomes fairly uniform along the dorsal‐ventral axis so measurement was at the mid ventricular layer (mid VL) at these stages; (J) SOX2‐positive cells per section were quantified at each stage (see Table S3). Error bars represent mean values ± SEM. A two‐sided unpaired Student's *t*‐test was used to determine whether differences in the mean values were statistically significant, ***P <* *0.001; **P <* *0.01; *P <* *0.05. Scale bars: 40 μm.

To characterise the changing dimensions and organisation of the ventricular layer in the developing mouse spinal cord, we delimited this cell population using immunofluorescence to detect SOX2‐positive neural progenitors and surrounding TUJ1‐expressing neurons. Measurements were made from embryonic day (E) 12.5 to E18.5 in the thoracic spinal cord (Fig. 1A–G’, Table S3).

At E12.5, the length of the ventricular layer spans the height of the transverse section of the spinal cord (615.2 ± 7.1 μm) and it is widest dorsally, tapering ventrally (Fig. 1A,H,I). At this stage, the apical surface of the neuroepithelium often meets at the midline, obscuring the central lumen in all but the dorsal region (Fig. 1A). The length of the ventricular layer is unchanged at E13.5; however, its dorsal width is now substantially reduced (from 85.6 to 33.6 μm or from 10 to 4 cell diameters; Fig. 1B,H,I). At this stage, the ventricular layer has an elongated rhomboid shape with the lumen open centrally (Fig. 1B). Here, in a nexus about five cell diameters below the roof plate, TUJ1‐positive neuronal processes are now visible at the dorsal midline and interdigitate between SOX2‐positive cells, which have lost their contiguous pseudostratified arrangement (Fig. 1B’). This local remodelling of the ventricular layer appears to be the first indication of the process of dorsal collapse.

By E14.5, the length of the ventricular layer is drastically reduced (to 387.1 ± 15.9 μm) due to dorsal collapse (Fig. 1C, H). Almost all of the dorsal region now contains interdigitated neuronal processes and more sparsely distributed SOX2‐positive cells (Fig. 1C’). Some of these more dorsal SOX2‐expressing cells now meet at the dorsal midline and the lumen has acquired an inverted tear‐drop shape (Fig. 1C’). This morphology, however, is quickly lost as dorsal collapse proceeds. Indeed, by E15.5 the ventricular layer is further reduced in length (200.1 ± 7.3 μm, or 15–20 cell diameters) and width, which becomes more uniform along the dorsal‐ventral axis (16.7 ± 2.2 μm, or 2 cell diameters), and only a narrow slit‐like lumen is apparent (Fig. 1D,D’,H,I). At E16.5, the length and width of the ventricular layer continue to diminish (Fig. 1E,E’,H,I) and some of the ventral‐most SOX2‐positive cells appear to dissociate from the ventricular layer (arrowheads in Fig. 1E’). Between E17.5 and E18.5, the ventricular layer is reduced in length just a few more cell diameters, as the more loosely arranged SOX2‐positive cells both dorsally and ventrally are further dispersed (110.4 ± 8.7 μm in length) (Fig. 1F–G’,H). The remaining contiguous SOX2‐expressing cell population that constitutes the ventricle appears now to have reached the final, oval‐shaped arrangement of what will be the future spinal cord central canal (Fig. 1G,G’).

These data identify large‐scale morphological changes and local cell re‐arrangements associated with ventricular layer attrition. These include, first, a reduction in dorsal width at E13.5 accompanied by a local loss of pseudostratified epithelial organisation, followed by dramatic reduction in length associated with widespread loss of epithelial organisation in the dorsal region and so dorsal collapse at E14.5. This is reflected in a significant reduction in the number of SOX2‐positive cells in the ventricular layer between E13.5 and E14.5 and between E14.5 and E15.5 (Fig. 1J). In addition, a later further attrition step, which we call here ventral dissociation, involves re‐arrangement of the ventral‐most SOX2‐positive cells, which now have nuclei located outside of the forming central canal lining.

### Reduced cell proliferation, but not apoptosis, accompanies ventricular layer attrition

**Figure 2:**
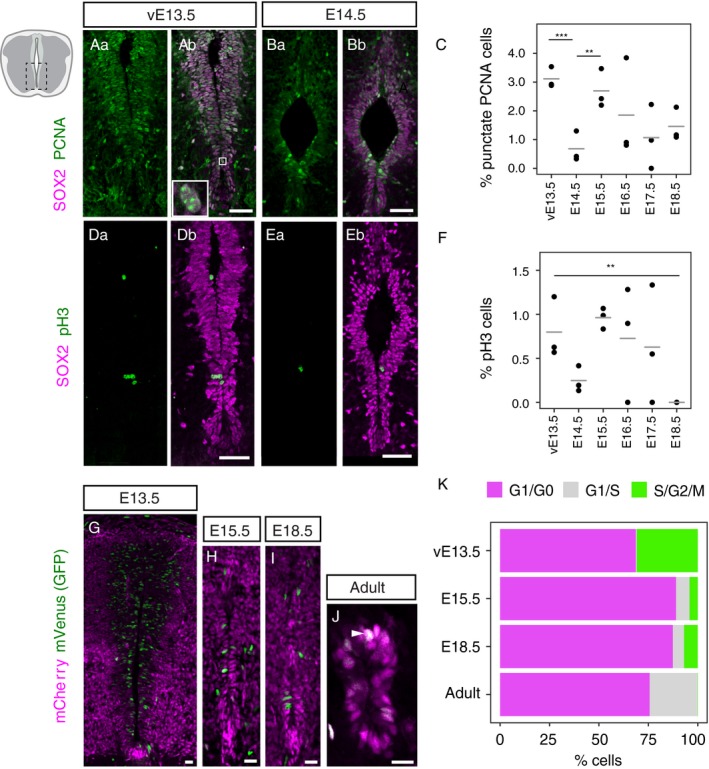
Cell proliferation declines as the spinal cord central canal is formed. (Aa–Ab) Immunofluorescence for the neural progenitor marker SOX2 (magenta) and the cell proliferation marker PCNA (green) in the ventral half of the ventricular layer at E13.5 and (Ba–Bb) at E14.5. Only cells with a punctuate PCNA pattern were counted in the analysis (white full box in Ab). (C) Percentage of SOX2‐positive cells with a punctate PCNA pattern in the spinal cord at E13.5–18.5. Each dot represents one embryo and the horizontal bars are the mean values of each stage. (Da–Db) Immunofluorescence for SOX2 (magenta) and the mitotic marker pH3 (green) in the ventral half of the ventricular layer at E13.5 and (Ea–Eb) E14.5. (F) Percentage of SOX2‐postive cells labelled with mitotic marker pH3 in the spinal cord at E13.5–18.5. Each dot represents one embryo and the horizontal bars are the mean values of each stage. (G) mCherry‐hCdt1 (magenta) and mVenus‐hGeminin (green) in the ventricular layer at E13.5, (H) E15.5, (I) E18.5 and (J) the adult central canal. (K) Percentage of cells in G1/G0 (magenta), G1/S (grey) and S/G2/M (green) in the ventral half of the ventricular layer at E13.5, the whole ventricular layer at E15.5 and E18.5, and in the adult central canal of Fucci2a mice. Asterisks show significant results from two‐sided, unpaired Student's *t*‐tests between each two correlative stages. ***P <* *0.001; **P <* *0.01; *P <* *0.05. Only statistically significant differences are indicated. The number of cells and sections analysed for each embryo and the exact *P*‐values for all comparisons can be found in Table S4 (for C), Table S5 (for F) and Table S6 (for K). Scale bars: (Aa–Eb) 40 μm, (G–J) 20 μm.

The reduction of SOX2‐positive cells in the ventricular layer as development proceeds, is at least in part accounted for by the delamination of cells from this proliferative zone to form neurons and, after E12.5, glial cells (Deneen et al. 2006; Stolt et al. 2003), including distinct astrocyte cell populations from E15.5 (Hochstim et al. 2008; Li et al. 2018). In addition, it is also possible that progenitor cells remaining in the ventricular layer reduce their proliferation rate and so cell replacement slows, accelerating the reduction of this progenitor cell population. To test the latter possibility, we set out to investigate cell proliferation parameters in the ventricular layer from E13.5 to E18.5. As it is largely the ventral half of the ventricular layer at E13.5 that is retained following dorsal collapse, we focused on this cell population and on the whole contiguous SOX2‐positive cell population from E14.5 onward (Fig. 2Aa–F). We first used the punctate pattern of PCNA, which indicates sites of DNA synthesis (Bravo & Macdonald‐Bravo, 1985), to determine the percentage of SOX2‐positive cells in S phase at each stage. This analysis revealed a reduction in the proportion of cells in S phase between E13.5 and E14.5 (Fig. 2Aa–C) and that thereafter this remains low and approximately constant to E18.5, apart from a slight increase at E15.5 (Fig. 2C). Next, the mitotic index was calculated by determining the percentage of phospho‐histone H3 (pH3)‐positive SOX2 cells at each stage. This revealed a similar pattern, in that the percentage of mitotic cells is low at E13.5 but lower at E18.5 (the mean differences between each two consecutive stages is not statistically significant, but the mitotic index at E18.5 is significantly lower than that at E13.5; *P *=* *0.017) (Fig. 2Da–F).

A limitation of the above approaches is the small number of cells observed with a punctate PCNA pattern or undergoing mitosis (Table S3). To extend this cell proliferation analysis, we therefore next assessed cell cycle phase distribution in ventricular layer cells using Fucci2a transgenic mice (Mort et al. 2014) at key stages: before (ventral E13.5) and after (E15.5) dorsal collapse, after ventral dissociation (E18.5) and in the adult central canal (Fig. 2G–J). These transgenic mice express two cell cycle‐regulated proteins fused to fluorescent proteins, one to mCherry (mCherry‐hCdt1) and the other to mVenus (mVenus‐hGeminin). This results in the accumulation of mCherry‐hCdt1 during the G1 and G0 phases of the cell cycle, making cells in G1 and G0 fluoresce red (shown here in magenta). mCherry‐hCdt1 is degraded at the G1/S transition following interaction with Geminin (Wohlschlegel et al. 2000; Tada et al. 2001) and mVenus‐hGeminin begins to accumulate in S, so cells in the G1/S transition fluoresce both red and green and appear yellow (shown here in white). mVenus‐hGeminin continues to be expressed during S/G2/M phases, making cells in these phases fluoresce bright green. The mVenus‐hGeminin is then rapidly degraded before cytokinesis (Mort et al. 2014; Sakaue‐Sawano et al. 2008). In agreement with our previous measurements, we found that the percentage of cells in S/G2/M decreases after E13.5 (from 30.8 ± 4.7% to 4.2 ± 0.5% E15.5, mean ± SEM, *P *=* *0.027) and remains relatively constant to E18.5 (6.9 ± 1.6%, *P *=* *0.239) (Fig. 2K). In the adult central canal, cells in S/G2/M are rarely detected (0.1 ± 0.1%, *P *=* *0.003). In contrast, the percentage of cells in the G1/G0 state increase from E13.5 to E15.5, from 68.7 ± 4.8% to 89.0 ± 1.9% (*P *=* *0.06), although the difference is not statistically significant. The percentage of cells in G1/G0 remains rather similar between E15.5 and E18.5 (87.4 ± 2.2%, *P *=* *0.39). This may reflect the intriguing emergence of a group of cells transitioning from G1 to S, which first becomes evident at late developmental stages (from 0.48 ± 0.24% at E13.5 to 6.8 ± 2.0% at E15.5, *P *=* *0.006) and increases still further in adult central canal cells (from 5.6 ± 1.6% at E18.5 to 24.4 ± 1.3%, *P *=* *0.0004) (Fig. 2K). This suggests that although the majority of adult central canal cells reside in G1/G0, the rest of this cell population now appears poised to progress through the cell cycle as they are beginning to upregulate Geminin (early S phase) and losing Cdt1 (Fig. 2J, K) (see Discussion). These findings show that after E13.5, gradually fewer cells progress through S/G2/M phases of the cell cycle and instead spend longer in G1. Our analysis suggests that this involves either arrest as cells exit the cell cycle into G0 or G1 lengthening, with about a quarter of adult cells being in a G1/S phase transition state. Together, these changes indicate a reduction in cell proliferation in the spinal cord ventricular layer as it transforms into the central canal lining.

A further cellular process that could contribute to the attrition of the ventricular layer is localised programmed cell death (apoptosis). This might operate in the dorsal region as the collapse commences from E13.5 and/or during subsequent refinement of the ventricular layer cell population as the central canal forms. To investigate this possibility, we carried out a TdT‐UTP nick end labelling (TUNEL) assay on transverse sections between E12.5 and E18.5 to detect DNA fragmentation, a hallmark of apoptosis. This revealed few apoptotic cells at any stage (Fig. S1A–G) and this was confirmed by immunofluorescence for activated caspase‐3 (Fig. S1H–O). These findings indicate that apoptosis does not contribute to ventricular layer attrition.

### Dissociation of a ventral‐most cell population contributes to central canal formation

**Figure 3:**
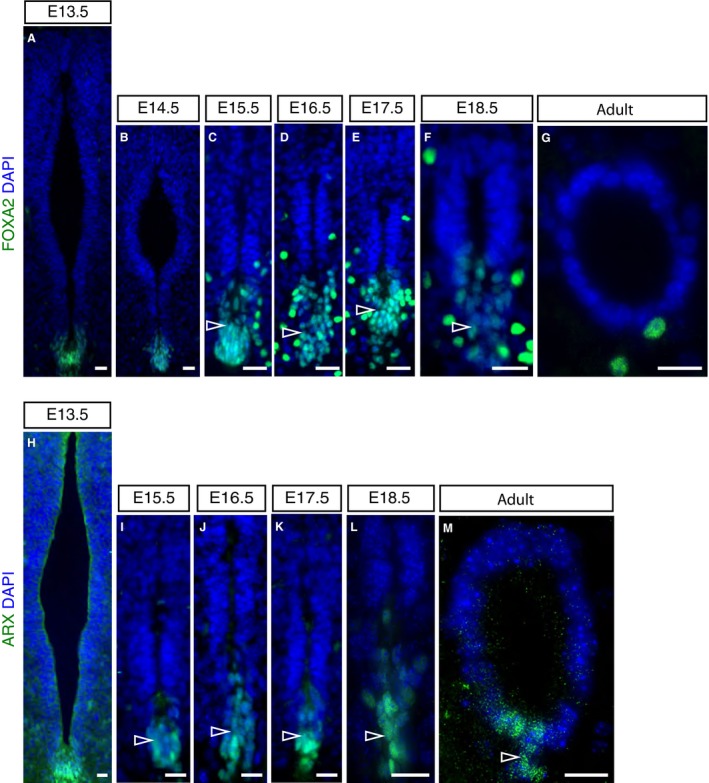
FOXA2 and ARX expression in the developing and adult mouse spinal cord. (A–G) Immunofluorescence for FOXA2 (green) at the indicated stages (E13.5: 33 sections, *n* = 3 embryos; E14.5: 8 sections, *n* = 2 embryos; E15.5: 18 sections, *n* = 5 embryos; E16.5: 22 sections, *n* = 5 embryos; E17.5: 16 sections, *n* = 4 embryos; E18.5: 26 sections, *n* = 4 embryos; adult (10‐ to 11‐week‐old mice): 18 sections, *n* = 5 mice). (H–M) Immunofluorescence for ARX expression (green) at the indicated stages (E13.5: 12 sections, *n* = 1 embryo; E15.5: 10 sections, *n* = 2 embryos; E16.5: 10 sections, *n* = 1 embryo; E17.5: 11 sections, *n* = 1 embryo; E18.5: 12 sections, *n* = 2 embryos; adult (10‐ and 24‐week‐old mice): 5 sections in each of *n* = 1 at each age. Dorsal is top, ventral is bottom. Nuclei are stained with DAPI (blue). Scale bars: 20 μm.

Analysis of the ventral‐most SOX2‐positive cells during development revealed a spatial rearrangement from E15.5, which culminated in the location of the nuclei of the majority of these cells outside the lining of the forming central canal (Fig. 1D–G’). To define further this ventral cell rearrangement and the cell populations involved, we analysed the expression of marker proteins of the ventral‐most cell population, the floor plate, during development. FOXA2 is a transcription factor that is initially localised in the floor plate and the adjacent p3 interneuron domain (Fig. 3A) (Cruz et al. 2010; Schafer et al. 2007). We found that this pattern of expression persists until E14.5 (Fig. 3B) and from E15.5, whereas ventral ventricular layer cells remain FOXA2‐positive, the ventral‐most cells begin to coalesce into a distinct group (arrowheads in Fig. 3C–F). In addition, a new group of cells that express FOXA2 highly, now also appear sub‐ependymally (outside the ventricle) (Fig. 3C–F). Moreover, although FOXA2 expression later is no longer detected in the ependymal cells lining the adult central canal, these FOXA2‐high sub‐ependymal cells remain (Fig. 3G) and may correspond to a subset of CSF‐contacting neurons (CSF‐cN”) (Petracca et al. 2016). *Arx* is a further well‐known floor plate marker from E9 to E12.5 (Miura et al. 1997; Cruz et al. 2010). A group of ARX‐positive cells continued to define this cell population at E13.5 (Fig. 3H). At later stages, ARX‐positive cells form an elongated arrangement, again with the nuclei of some cells remaining in the ventral ventricular layer and other nuclei located more ventrally to these (arrowheads in Fig. 3I–L). In contrast with FOXA2 expression, some ARX‐positive cells then persist in the adult ventral central canal lining, as noted previously (Khazanov et al. 2017) (Fig. 3M). These patterns of expression corroborate the dissociation of ventral‐most floor plate cells observed by monitoring the changing arrangement of SOX2‐expressing cells and support the inclusion of a subset of floor plate cells within the walls of the adult central canal.

**Figure 4:**
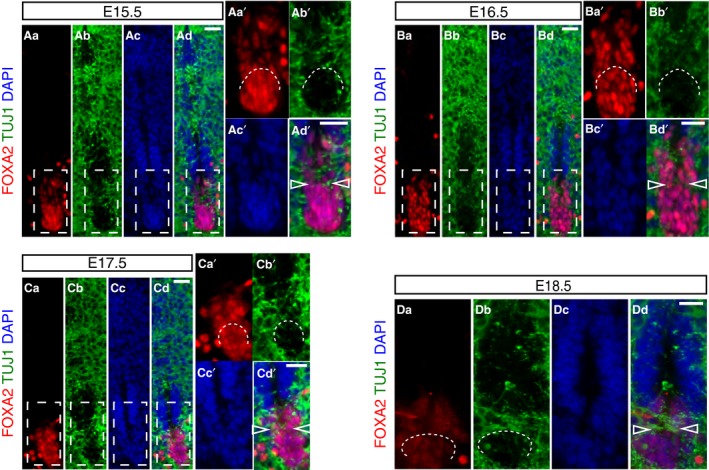
Ventral cell rearrangement during late mouse spinal cord development. (Aa–Dd) Immunofluorescence for FOXA2 (red) and TUJ1 (green) in mouse spinal cord development at stages indicated (E15.5: 11 sections, *n* = 3 embryos; E16.5: 10 sections, *n* = 3 embryos; E17.5: 9 sections, *n* = 3 embryos; E18.5: 9 sections, *n* = 2 embryos). Higher magnification of regions outlined in white dashed line boxes are shown in Aa’‐Ad’, Ba’‐Bd’ and Ca’‐Cd’. Partition of the ventral‐most floor plate cell population by TUJ1‐positive neuronal processes, indicated by curved white dashed lines and by arrowheads in merged images. Nuclei are stained with DAPI (blue). Scale bars: 20 μm.

**Figure 5:**
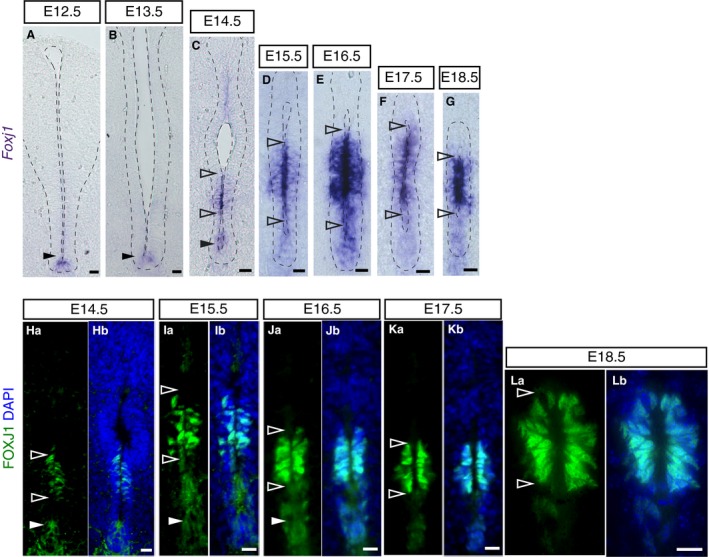
Foxj1 mRNA and protein expression during mouse spinal cord development. (A–G) *In situ* hybridisation showing the dynamic expression of *Foxj1* mRNA at the indicated stages (E12.5: 22 sections, *n* = 5 embryos; E13.5: 32 sections, *n* = 7 embryos; E14.5: 32 sections, *n* = 7 embryos; E15.5: 33 sections, *n* = 12 embryos; E16.5: 86 sections, *n* = 15 embryos; E17.5: 114 sections, *n* = 18 embryos; E18.5: 94 sections, *n* = 11 embryos). Note apical localisation of transcripts. Nuclei are stained with DAPI (blue). (Ha–Lb) Immunofluorescence showing dynamic expression of the FOXJ1 protein (green) at indicated stages (E14.5: 66 sections, *n* = 4 embryos; E15.5: 33 sections, *n* = 3 embryos; we also confirm findings of Li et al. 2018 at later stages E16.5: 12 sections, *n* = 1 embryo; E17.5: 12 sections, *n* = 1 embryo; E18.5: 12 sections, *n* = 1 embryo). Nuclei are stained with DAPI (blue). Note that low levels/gaps in expression are found in the ventricular layer between the region closer to the dorsal lumen and the domain adjacent to the floor plate at E14.5 (arrowheads in C and Ha). Scale bars: (A,B) 40 μm, (C–Lb) 20 μm.

To elucidate further the remodelling of this ventral cell population, we next combined FOXA2 and TUJ1 immunofluorescence in order to visualise the relationship between FOXA2‐positive cell groups and neuronal processes. This revealed TUJ1‐positive cell processes intervening between the cells lining the central canal and the dissociating ventral‐most cell group from E15.5, with a pronounced ventral commissure apparent by E18.5 (Fig. 4Aa–Dd). Intriguingly, this dissociation of the ventral‐most cell population occurs concomitant with a change in the expression pattern of *Foxj1*, the master regulator of the ciliogenesis programme (Choksi et al. 2014) and a mediator of migratory cell behaviour of spinal cord ependymal cells in response to injury (Li et al. 2018). *Foxj1* transcript and protein levels decline in the floor plate as ventral dissociation begins at E15.5 (compare ventral region in Fig. 5A–D and Ha–Ib) but is upregulated in ventral progenitors from E14.5 (Fig. 5C–G and Ha–Lb) and is expressed in the majority of cells lining the central canal, although only at a low level in ventral cells at E18.5 (Li et al. 2018). Together, these observations identify a distinct dissociation event which resolves the ventral ventricular cell population into two anatomically distinct groups: those with nuclei within the central canal lining and a cell group whose nuclei are now located more ventrally outside the canal.

### A floor plate contribution to the spinal cord central canal lining is supported by analysis of Shh pathway components, which also reveals a gradual decline in Shh signalling

The presence of ARX‐positive cells in the ventral pole of the central canal supports the notion that some floor plate‐derived cells contribute to this structure. Indeed, lineage tracing of cells expressing the floor plate marker *Nato3* (*Ferd3l*), using a Nato3‐LacZ line, revealed some LacZ‐positive cells in this region (Khazanov et al. 2017). Further, the regulatory relationships between *Foxa2*,* Nato3* and Shh signalling that underpin floor plate maturation (Cruz et al. 2010; Mansour et al. 2011, 2014), suggest that the Shh pathway is active as the central canal forms (Rowitch et al. 1999). Moreover, as Shh acts as a mitogen as well as a morphogen in the early neural tube it is interesting to assess whether cells retaining floor plate markers continue to act as a signalling centre during mid‐late development and postnatally.

**Figure 6:**
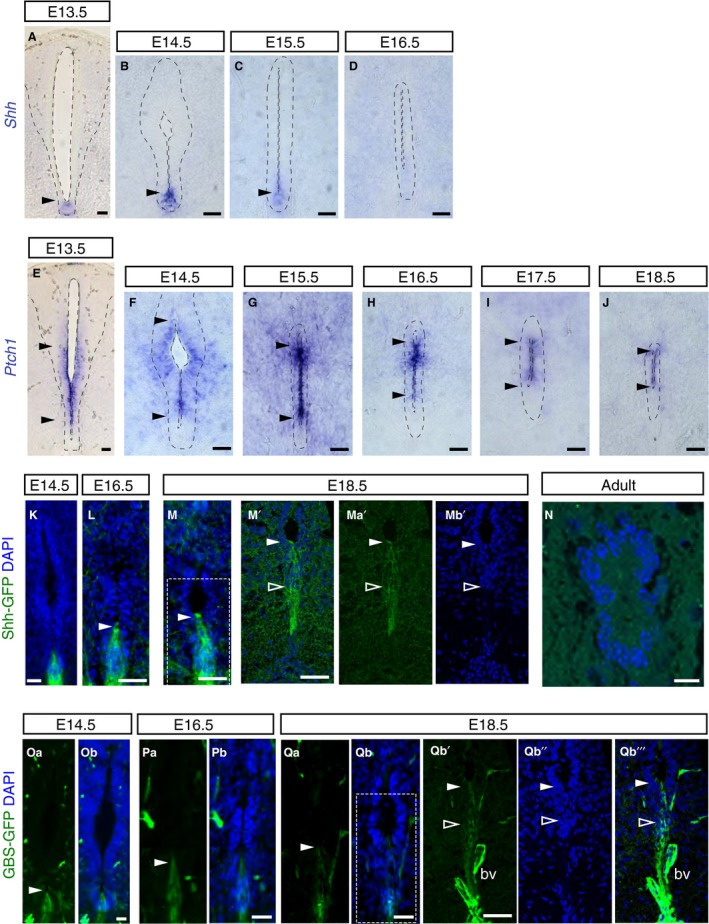
Shh activity persists after decline in *Shh* transcripts during mouse spinal cord development. (A–D) *In situ* hybridisation showing *Shh* mRNA expression in the floor plate (arrowheads) at the indicated stages (E13.5: 14 sections, *n* = 3 embryos; E14.5: 15 sections, *n* = 5 embryos; E15.5: 58 sections/explants, *n* = 9 embryos; E16.5: 38 sections/explants, *n* = 9 embryos). From E17.5 onwards, *Shh* mRNA expression was no longer detected in the spinal cord (E17.5: 38 sections/explants, *n* = 8 embryos; E18.5: 18 sections, *n* = 3 embryos; 24‐ and 40‐week‐old: 17 sections, *n* = 3 adult mice; data not shown). (E–J) *Ptch1* mRNA expression at the indicated stages (E13.5: 27 sections, *n* = 5 embryos; E14.5: 26 sections/explants, *n* = 5 embryos; E15.5: 43 sections/explants, *n* = 6 embryos; E16.5: 49 sections/explants, *n* = 8 embryos; E17.5: 40 sections/explants, *n* = 8 embryos; E18.5: 16 sections, *n* = 3 embryos). Note *Ptch1* is excluded from the floor plate and detected in ventral half of the ventricular layer (between arrowheads) and persists in the forming central canal, but was no longer detected in adult (40‐week‐old: 24 sections/explants, *n* = 3 mice; data not shown). (K–Mb’’’) Immunofluorescence for GFP in Shh‐GFP embryos was detected in the floor plate during development (arrowheads) including dissociating ventral cell population at later stages (outlined white arrowhead): E14.5: 17 sections, *n* = 1 embryo; E16.5: 17 sections, *n* = 1 embryo; E18.5: 18 sections, *n* = 1 embryo, (M’‐Mb’ single z‐plane confocal images of region shown in white dashed‐line square in M and more ventral domain) (*n*) but not in adult central canal (9–10 week adult (88 sections, *n* = 3 mice); (Oa–Qb’’’). Immunofluorescence for GFP (arrowheads) in GBS‐GFP embryos revealed positive cells in the ventral region at indicated stages (E14.5: 47 sections, *n* = 3 embryos; E16.5: 43 sections, *n* = 3 embryos; E18.5: 33 sections, *n* = 3 embryo) (Qb’–Qb’’’ single z‐plane confocal images of region shown in white dashed‐line square in Qb and more ventral domain). K–M’, Mb’, *n*, Ob, Pb, Qb qb’’, Qb’’’ nuclei are stained with DAPI (blue). bv, auto‐fluorescent blood vessel. Scale bars: 40 μm, except *n*: 10 μm.

Using *in situ* hybridization, we found that transcripts for *Shh* in the floor plate were attenuated at E15.5 and undetectable by E16.5 (Fig. 6A–D). In contrast, transcripts for the Shh receptor and transcriptional target *Ptch1* (Goodrich et al. 1997; Marigo & Tabin, 1996; Pearse et al. 2001; Vokes et al. 2008) persisted for longer in the presumptive central canal lining but were much reduced by E18.5 (Fig. 6E–J). These observations suggest that Shh signalling persists after *Shh* transcription ceases, but declines as the central canal forms. We investigated this further by looking at GFP expression in two Shh signalling reporter mouse lines: one expressing a tagged Shh‐GFP transgene (Chamberlain et al. 2008) and another expressing Gli‐binding site motifs linked to GFP (GBS‐GFP), which provides a further readout of Shh signalling activity (Balaskas et al. 2012). Expression of Shh‐GFP was restricted to the floor plate at all embryonic stages examined including E18.5 (Fig. 6K–Mb’). This persistence of Shh‐GFP compared with that of *Shh* transcripts may be indicative of residual Shh protein (consistent with continued *Ptch1* expression), but could also reflect a longer half‐life of the GFP tagged protein. Ependymal cells of the adult central canal, however, lacked any Shh‐GFP (Fig. 6N).

The presence of cytoplasmic GFP in these Shh‐GFP embryos further allowed us to observe changing cell morphologies and revealed the elongation of cells associated with the ventral dissociation event (Fig. 6K–M). Moreover, some GFP‐expressing cell processes remain integrated within the apical surface of the ventricle even at late stages (Fig. 6M–Mb’); this may reflect inclusion of floor plate‐derived cells in the central canal lining and/or additional retained contact by some or all cells in the ventral‐most dissociating cell group.

GBS‐GFP was expressed more widely than Shh‐GFP and was detected in the floor plate and adjacent ventricular layer cells at E14.5 (Fig. 6Oa–Ob). However, it also became restricted to the floor plate at subsequent stages, with a dwindling number of expressing cells continuing to contact the ventricular lumen at E18.5 (Fig. 6Oa–Qb’’’). These data indicate that Shh signalling persists locally for some days after *Shh* transcription ceases at E14.5, declining slowly as the central canal forms and appears lost in the adult.

### Dorsal BMP signalling persists and extends ventrally during central canal formation

The roof plate origin of dorsally located radial glia‐like cells that are integrated into the central canal lining during dorsal collapse was recently confirmed in mice (Xing et al. 2018; Shinozuka et al. 2019). During development, roof plate cells express Wnt and BMP ligands, which act in opposition to Shh signalling (le Dreau & Marti, 2012). Moreover, BMP signalling has been implicated in the regulation of quiescence and proliferation of adult brain neural stem cells (Llorens‐Bobadilla et al. 2015; Mira et al. 2010).

**Figure 7:**
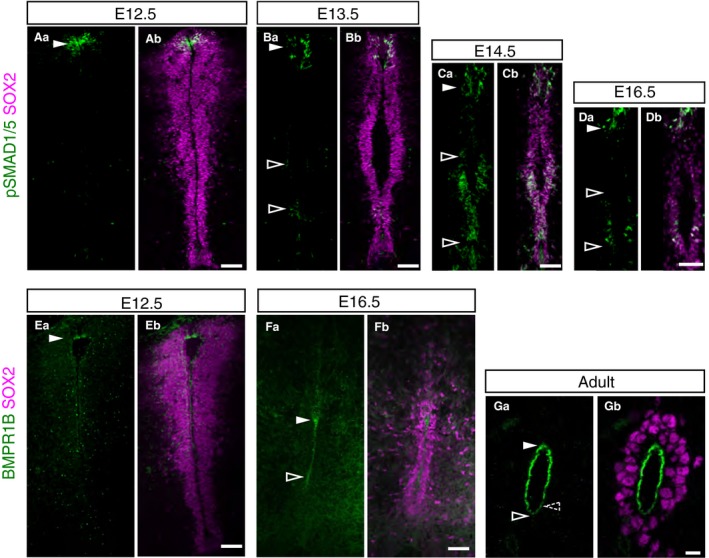
Expanding BMP signalling and BMPR1B expression during mouse spinal cord development. (Aa–Bb) Immunofluorescence for phospho‐SMAD1/5 (green) detected in a dorsal cell population at E12.5 (16 sections, *n* = 2 embryos) and E13.5 (16 sections, *n* = 3 embryos) (full arrowheads), phospho‐SMAD1/5 was also detected in the ventral ventricular layer at E13.5 (outlined arrowhead); (Ca–Db). At E14.5 (36 sections, *n* = 3 embryos) and E16.5 (14 sections, *n* = 2 embryos), phospho‐SMAD1/5 was detected in dorsal (full arrowheads) and ventral regions of the ventricular layer (outlined arrowheads). (Ea–Fb) BMPR1B (green) was enriched dorsally at E12.5 (10 sections, *n* = 1 embryo) (full arrowhead) and was detected dorsally and apically in ventricle abutting cells (between arrowheads) at E16.5 (23 sections, n = 3 embryos). (Ga,Gb) Pronounced apical localisation of BMPR1B in adult central canal (10‐ to 11‐week‐old mice: 22 sections, *n* = 2 mice), which was more weakly detected ventrally (dashed outline arrowhead). Progenitor cells within the ventricular layer are labelled with SOX2 (magenta). Scale bars: 50 μm, except Adult: 10 μm.

To investigate how BMP signalling changes as the central canal is formed we carried out immunofluorescence against phosphorylated SMAD1/5 (pSMAD1/5), a readout of BMP signalling, and the BMP receptor BMPR1B. Analysis of patterns of pSMAD1/5 detection revealed that BMP activity is restricted to roof plate cells at E12.5 and persists in SOX2‐positive cells located dorsally until at least E16.5 (Fig. 7Aa–Db). However, from E13.5, pSMAD1/5 was additionally detected in a ventral domain (Fig. 7Ba–Bb), which became more widespread at E14.5 (Fig. 7Ca–Cb) and continued to be detected dorsally and ventrally at E16.5 (Fig. 7Da–Db). Analysis of BMPR1B expression revealed that this was present in the roof plate at E12.5 (Fig. 7Ea–Eb) and that this persisted and extended ventrally at E16.5, where the protein was enriched apically (Fig. 7Fa–Fb). This pattern of expression continued in the adult central canal lining, with low‐level protein detected ventrally (Fig. 7Ga–Gb). These findings indicate that BMP signalling is dynamically regulated in the developing spinal cord: initial dorsally restricted activity spreads to ventral regions and this is mirrored in the changing expression pattern of BMPR1B.

## Discussion

**Figure 8:**
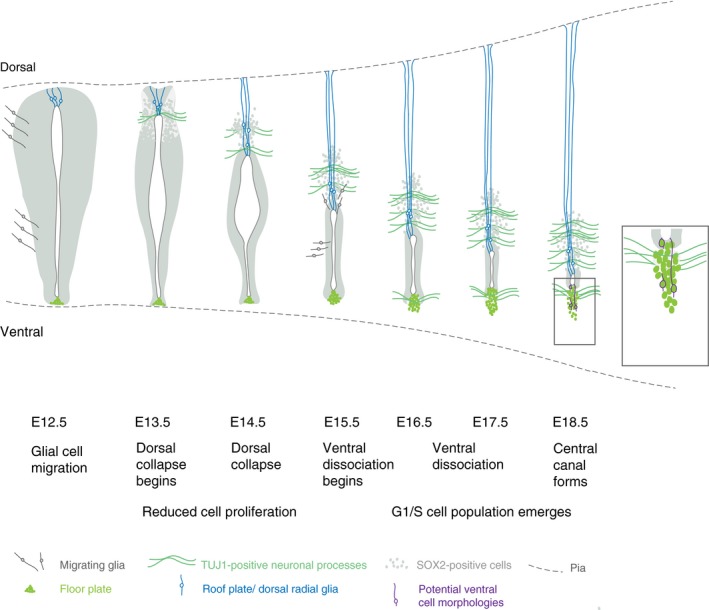
Summary of steps contributing to spinal cord ventricular layer attrition. Schematic of key changes in cell arrangement and cell cycle in the spinal cord ventricular layer at daily intervals leading up to the formation of central canal. SOX2‐positive ventricular cells are indicated in grey (note that floor plate cells are also SOX2‐positive, but distinguished here in bright green, and SOX2 is also expressed in astrocytes at later stages). Glial cell migration from the ventricular layer begins prior to dorsal collapse (Deneen et al. 2006; Stolt et al. 2003) and distinct glia cell populations continue to emerge from the ependymal cell population forming the central canal, including dorsal and lateral/ventral astrocytes from E15.5 (Hochstim et al. 2008; Li et al. 2018). Dorsal collapse is accompanied by elongation of roof plate cells, which give rise to dorsal radial glia (in blue) (Xing et al. 2018; Shinozuka et al. 2019), appearance of the dorsal commissure (TUJ1‐positive cell processes) and reduced proliferation of ventricular layer cells (this study). These events are followed by dissociation of ventral‐most floor plate cell group (the nuclei of these cells are not included in the lining of forming central canal, but some of these cells may retain a contact with its apical surface, potential ventral cell morphologies are indicated in purple), accompanied by appearance of the ventral commissure (TUJ1‐positive cell processes) and an increase in the percentage of cells in G1/S phase of the cell cycle (this study).

This study provides evidence for multiple steps that contribute to the reduction of the spinal cord ventricular layer during development and lead ultimately to the formation of the adult spinal cord central canal. These include initial local loss of ventricular layer organisation below the roof plate, which spreads as dorsal collapse proceeds; a reduction in cell proliferation, involving an increase in the percentage of cells in the G1/S phase of the cell cycle; and cell rearrangement in the floor plate, involving dissociation of the ventral‐most cell population. Despite this ventral remodelling, gene expression patterns supported previous reporter line studies indicating incorporation of some floor plate cells into the adult central canal lining and the emerging view that this ependymal cell population has a heterogeneous origin. Moreover, analysis of key signalling pathways revealed spread of dorsal and decline in ventral signalling in this developing ventricular layer. Together these findings identify distinct processes that contribute to formation of the adult spinal cord central canal (summarised in Fig. 8).

We show that ventricular layer attrition begins not long after the switch from neurogenesis to gliogenesis in the developing spinal cord and is first manifested by the narrowing and local disorganisation of the dorsal ventricular layer at E13.5. The disruption of the close alignment of SOX2‐expressing progenitor cells below the roof plate appears to be the initiation site for dorsal collapse, which progresses rapidly in subsequent days. The appearance of neuronal cell processes between these progenitor cells and across the midline, may equate to the initiation of the dorsal commissure, which is more apparent at later stages in this position (Comer et al. 2015). It is interesting that the novel cell rearrangement we observe later in ventral‐most regions from E15.5, named here as ventral dissociation, is also associated with the appearance of neuronal cell processes that traverse the midline, here to form the ventral commissure (Comer et al. 2015). In this case, the appearance of axons crossing the midline appears to separate those cells with nuclei in the central canal from those with nuclei now located more ventrally, which may nevertheless still contact the lumen (Fig. 8). Indeed, the ventral extension of this cell population is also evident in Foxj1‐GFP transgenic embryos (Li et al. 2018). These observations raise the possibility that commissure establishment is one of the mechanisms by which the ventricular layer is restricted in ventral as a well as dorsal regions.

Studies at earlier stages in the mouse embryo (between E9.5 and E11.5) have shown that the cell proliferation rate in the ventricular layer declines during neurogenesis, with little difference between dorso‐ventrally distinct progenitor cell populations (Kicheva et al. 2014). Our finding of a reduction in the proportion of cells in S and M phases of the cell cycle at later stages suggests that this decline in proliferation continues and contributes to ventricular layer attrition after E13.5. Intriguingly, analysis of the cell cycle using Fucci2a reporter mice uncovered a progressive increase in cells in G1/S from E15.5, which ultimately accounted for almost a quarter of the cells in the adult central canal lining. As cells are asynchronously distributed across the cell cycle, this could be attributed to G1 lengthening: with more cells in G1 resulting in an increase in cells transitioning from G1 to S phase at a given time. Another intriguing possibility is that ventricular cells progressively switch to a cell cycle variant known as endocycle as they differentiate into ependymal cells. Endocycling cells go through G1 and S but do not undergo mitosis, becoming polyploid (Edgar et al. 2014). This would be consistent with the progressive increase in the percentage of cells G1/S and the decrease in S/G2/M cells we detected in Fucci2a embryos and mice. Indeed, in other studies using Fucci reporters, endoreplicating cells seem to co‐express Geminin and Cdt1 (and thus appear yellow) for longer (Cao et al. 2017; Lazzeri et al. 2018). A switch to endocycling would potentially lead to higher genomic output, which might then enhance the secretory function of these cells. A further possibility is that the overall decline in cell proliferation is due to an increase in the cells that enter the quiescent state (G0) in G1. However, it is difficult to distinguish cells in G1 or G0 using the Fucci2a system, as they both appear red. Whether the movement of cells into a reversible G0 state explains why adult central canal cells are mostly quiescent but can readily re‐enter the cell cycle in response to multiple extrinsic stimuli or if G1/S cells also contribute to the reactivity of this cell population remains to be investigated. The latter is certainly an intriguing cell population that is not apparent in post‐mitotic brain ependymal cells analysed in the Fucci2a mouse (Ford et al. 2018).

Early expression pattern studies in a range of amniote embryos suggest that the progenitor cell population expressing *Pax6* and *Nkx6‐1*, but not *Nkx2‐2*, is retained during development and gives rise to the ependymal cells of the adult central canal (Fu et al. 2003; Yu et al. 2013). Accumulating evidence, however, shows that the origin of these cells is rather heterogeneous, including cells derived from the roof plate (Xing et al. 2018; Shinozuka et al. 2019) and from the floor plate (Khazanov et al. 2017; Ghazale et al. 2019). Here, we found that cells expressing the floor plate marker ARX, but not FOXA2, are present within the lining of the central canal and that Shh‐GFP expressing cells are found integrated into this cell population at late embryonic stages.

An outstanding question is whether cells derived from the roof plate and floor plate remain as signalling centres during late embryonic development and into the adult spinal cord, as they do in regenerative species such as the axolotl and zebrafish (Schnapp 2005, Reimer 2009). Here, we have shown that Shh signalling declines from the ventral midline/floor plate at late embryonic stages and that Shh‐GFP is no longer detected in central canal cells of the adult spinal cord. While it is possible that the detection of GFP in Shh‐GFP and GBS‐GFP transgenic embryos reflects the relatively long half‐life of GFP rather than persistence of SHH, the detection of Shh targets *Ptch1* (this study) and *Gli* (Yu et al. 2013) supports the possibility of dwindling Shh signalling at these late stages. This would also be consistent with studies using transgenic mice to delete *Shh* from the floor plate or its mediator *Smo* from the neural tube (Yu et al. 2013), as well as *Gli2* mutants (Ding et al. 1998; Matise et al. 1998), all of which fail to form a proper central canal, although confirmation of a persisting role for Shh signalling requires interference with this pathway specifically at late stages or in the adult.

In contrast, we found that the activity of the BMP signalling pathway, which opposes Shh signalling in the developing spinal cord, although initially dorsal, expands as the central canal forms, with pSMAD1/5 detected extensively in more ventral regions as well as in dorsal roof plate‐derived cells. This is further reflected by BMPR1B expression, which was detected in all ependymal cells of the adult central canal, albeit at a lower level in the most ventral cells. Recent studies have shown that Wnt signalling first from roof plate/dorsal radial glia cells and later also from the ependymal cells is required to maintain the ependymal epithelium (Xing et al. 2018; Shinozuka et al. 2019), and Wnt promotion of BMP signalling (found at earlier stages, Ille et al. 2007) would be further consistent with the expansion of dorsal signalling. Taken together, this investigation of these key signalling pathways in the late embryonic and adult spinal cord suggests that dorsal signalling comes to prevail in cells of the ventricular layer as the central canal forms and ependymal cells differentiate. The involvement of BMP signalling in the regulation of adult neural stem cell quiescence in the brain (Llorens‐Bobadilla et al. 2015; Mira et al. 2010) now prompts further experiments to investigate whether this spread of BMP activity promotes acquisition of the quiescent cell state, including changes in cell cycle, and thereby sets aside the elusive spinal cord neural stem cell as the central canal forms.

## Funding

M.A.C. was supported by a studentship from the Anatomical Society of Great Britain and Northern Ireland, awarded to P.F. and K.G.S. and for a short period by the ISSF (WT204816/Z/16). K.G.S. is a Wellcome Trust Investigator (WT102817AIA). A.R.A. is supported by the European Union's Horizon 2020 research and innovation programme under the Marie Skłodowska‐Curie grant agreement No. 753812 and G.S. by a Wings for Life project grant (WFL‐UK‐24/17‐ Prj 171). N.S. is a masters and MG an undergraduate student at the University of Dundee. Imaging work was further supported by a Wellcome multi‐user equipment grant (WT 208401).

## Competing interests

The authors declare that they have no competing interests.

## Author contributions

K.G.S. and P.F. designed the project. M.A.C., G.S., N.S. and M.G. designed and performed experiments. M.A.C., A.R.A. and K.G.S. interpreted the data and wrote the paper. All authors read and approved the final manuscript.

## Supporting information


**Figure S1.** Analysis of programmed cell death during mouse spinal cord development.

**Table S1.** Primary and secondary antibodies.
**Table S2** Plasmids, restriction endonucleases and RNA polymerases.

**Table S3.** Length and width measurements of the spinal cord ventricular layer in mouse embryos from embryonic day (E) 12.5 to E18.5.

**Table S4.** Counts of SOX2‐positive and double SOX2‐positive proliferating cell nuclear antigen (PCNA)‐positive cells in the spinal cord ventricular layer from embryonic day (E) 13.5 to E18.5.

**Table S5.** Counts of SOX2‐positive cells and double SOX2‐ phospho‐histone H3 (pH3)‐positive cells in the spinal cord ventricular layer from embryonic day (E) 13.5 to E18.5.

**Table S6.** Two‐sided, unpaired Student's *t*‐test with pooled standard deviation and equal variance.
