# Supplementary material for: Multiple steps characterise ventricular layer attrition to form the ependymal cell lining of the adult mouse spinal cord central canal
Source: J Anat. 2019 Oct 31;236(2):334–50. doi: 10.1111/joa.13094 (PMC6956438; doi:10.1111/joa.13094)
Supplement: Supplementary file 2 — Table S1. Primary and secondary antibodies. Table S2 Plasmids, restriction endonucleases and RNA polymerases. [file JOA-236-334-s002.docx]

**Table S1**

Primary and secondary antibodies

Primary antibodies

rabbit anti-ARX (1:1000, gift from Dr J. Chelly, IGBMC, Paris, France),

mouse anti-BMPR1B (1:50, Santa Cruz Biotechnology sc-515886),

mouse anti-CASPASE 3 (1:400, Abcam, ab13585),

rabbit anti-FOXA2 (1:200, Abcam, ab108422),

rabbit anti-FOXJ1 (1:200, Sigma, HPA005714),

chicken anti-GFP (1:400, Abcam, ab13970),

goat anti-GFP (1:400, Abcam, ab6673),

mouse anti-PCNA (1:200, Merck Millipore, MAB424),

rabbit anti-pH3 (1:400, CST, 9713),

rabbit anti-pSMAD1/5 (1:50, CST, 9516),

goat anti-SOX2 (1:200, Immune System, GT15098),

rabbit anti-SOX2 (1:200-1:2000, Merck Millipore),

mouse anti-TUJ1 (1:500, Covance Research Products Inc, MMS-435P),

rabbit anti-TUJ1 (1:1000, Sigma, T2200).

Secondary antibodies

All secondary antibodies were Alexa Fluor secondary antibodies from Thermo Fisher Scientific and diluted 1:500.

Alexa 488 Donkey anti-rabbit IgG (H+L) Cat. # A21206;

Alexa 568 Donkey anti-rabbit IgG (H+L) Cat. # A10042;

Alexa 568 Goat anti‐rabbit IgG (H+L) Cat. # A11011;

Alexa 594 Donkey anti‐goat IgG (H+L) Cat. # A11058;

Alexa 594 Donkey anti-rabbit IgG (H+L) Cat. # A21207;

Alexa 594 Goat anti-rabbit IgG (H+L) Cat. # A11012;

Alexa 647 Donkey anti‐goat IgG (H+L) Cat. # A21447.

Alexa 647 Donkey anti‐mouse IgG (H+L) Cat. # A31571.

**Table S2**

Plasmids, restriction endonucleases and RNA polymerases

| **Plasmid – Gene product** | **Restriction endonucleases** | **RNA Polymerases** |
| --- | --- | --- |
| pYX-ACS-mFoxJ1 (full-length) | Anti-sense: SalI (Roche, Cat. # 10567663001) | T3 (Roche, Cat. # 11031171001) |
|  | Sense: Not1 (Roche, Cat. # 11014714001) | T7 (Roche, Cat. # 10881775001) |
| pCIG-mPtch1 (full-length) | SpeI (Roche, Cat. # 11008943001) | T3 |
| pBluescript II SK-mShh (insert size: 640 bp) | Anti-sense: HindIII | T3 |
|  | Sense: Not1 | T7 |

**Tables S3-S6** are Excel spread sheets (provided separately).
